# Intra-operative spectroscopic assessment of surgical margins during breast conserving surgery

**DOI:** 10.1186/s13058-018-1002-2

**Published:** 2018-07-09

**Authors:** Dustin W. Shipp, Emad A. Rakha, Alexey A. Koloydenko, R. Douglas Macmillan, Ian O. Ellis, Ioan Notingher

**Affiliations:** 1 School of Physics and Astronomy, University of Nottingham, Nottingham, NG7 2RD UK; 2 Division of Oncology, School of Medicine, University of Nottingham, Nottingham, NG5 1PB UK; 3 Mathematics Department, Royal Holloway, University of London, Egham, TW20 0EX UK; 4 Nottingham Breast Institute, Nottingham University Hospitals NHS Trust, Nottingham, NG5 1PB UK

**Keywords:** Auto-fluorescence, Breast cancer, Raman spectroscopy, Intra-operative margin evaluation

## Abstract

**Background:**

In over 20% of breast conserving operations, postoperative pathological assessment of the excised tissue reveals positive margins, requiring additional surgery. Current techniques for intra-operative assessment of tumor margins are insufficient in accuracy or resolution to reliably detect small tumors. There is a distinct need for a fast technique to accurately identify tumors smaller than 1 mm^2^ in large tissue surfaces within 30 min.

**Methods:**

Multi-modal spectral histopathology (MSH), a multimodal imaging technique combining tissue auto-fluorescence and Raman spectroscopy was used to detect microscopic residual tumor at the surface of the excised breast tissue. New algorithms were developed to optimally utilize auto-fluorescence images to guide Raman measurements and achieve the required detection accuracy over large tissue surfaces (up to 4 × 6.5 cm^2^). Algorithms were trained on 91 breast tissue samples from 65 patients.

**Results:**

Independent tests on 121 samples from 107 patients - including 51 fresh, whole excision specimens - detected breast carcinoma on the tissue surface with 95% sensitivity and 82% specificity. One surface of each uncut excision specimen was measured in 12–24 min. The combination of high spatial-resolution auto-fluorescence with specific diagnosis by Raman spectroscopy allows reliable detection even for invasive carcinoma or ductal carcinoma in situ smaller than 1 mm^2^.

**Conclusions:**

This study provides evidence that this multimodal approach could provide an objective tool for intra-operative assessment of breast conserving surgery margins, reducing the risk for unnecessary second operations.

**Electronic supplementary material:**

The online version of this article (10.1186/s13058-018-1002-2) contains supplementary material, which is available to authorized users.

## Background

Breast conserving surgery (BCS), also referred to as lumpectomy or wide local excision, is currently the most widely used surgical procedure for resection of breast cancer [1]. The goal of BCS is to remove the entire tumor while leaving healthy breast tissue intact, providing better cosmetic outcome. Nevertheless, this is challenging because of the lack of tools available for intra-operative assessment of margins to indicate complete tumor excision.

Postoperatively, typically over a period of 1–2 weeks, the excised tissues are examined histologically to determine the proximity of tumor to the surface of the excision. In more than 20% of BCS procedures, positive margins are detected (i.e. tumor close to the edge) and additional operations are required to achieve complete excision [2, 3]. Nearly half of these “re-excisions” are for “on-ink” margins [3], meaning that tumor was found on the surface of the excised tissue. Guidelines from the Society of Surgical Oncology and the American Society for Radiation Oncology state that clear on-ink margins are sufficient to remove tumor and more widely clear margins did not significantly increase the risk of recurrence [4, 5].

Intra-operative resection of additional tissue (i.e. cavity shaves) has been shown to reduce the need for re-excisions [6]. However, cavity shaving can result in excessive tissue loss and poor cosmetic outcomes. Additional techniques are therefore needed to assess the margins of BCS specimens within intra-operative timescales (i.e. less than 30 min). Frozen section histopathologic assessment and cytologic imprint preparation (i.e. touch preparation) analysis can assess margins within this time [7, 8], but are often considered impractical for BCS due to the large size of the specimens, sampling errors [7, 9], and sample preparation artifacts [10] in addition to pathologist time and cost implications. The MarginProbe device, which assesses margins using radiofrequency spectroscopy, has entered operating theaters, but with 75.2% sensitivity and 46.4% specificity [11]. ClearEdge measures tissue-specific electrical properties with preliminary results indicating sensitivity of 84.3–87.3% and specificity of 81.9–75.6% [12]. Higher diagnostic accuracy has been reported for techniques with higher molecular specificity, such as fluorescence lifetime imaging (FLIm) [13] and mass spectrometry [14]. A recent preliminary study using a FLIm probe on 2 × 2 cm^2^ cut breast tissues indicted automated classification accuracy greater than 97% [13]. For mass spectrometry handheld devices, 93.4% sensitivity and 94.9% specificity were reported, but with spatial resolution limited to approximately 4 × 4 mm^2^ [15]. Limitations related to spatial resolution and tissue sampling coverage make hand-held technologies vulnerable to missing small tumors (e.g. ductal carcinoma in situ (DCIS) smaller than 1 mm^2^), which are responsible for a disproportionate number of re-excisions [5].

Sampling errors may be overcome by optical imaging techniques that can provide diagnosis with microscopic spatial resolution [16–22]. While diagnoses with sensitivity and specificity as high as 93% have been reported, structure-based imaging diagnoses rely on specially trained pathologists and are therefore subject to inter-observer and intra-observer variability, especially if large, detailed images need to be viewed [19, 21]. Attempts to avoid subjectivity through automated diagnosis by diffuse reflectance spectroscopy (DRS) [23, 24], elastic scattering spectroscopy (ESS) [25], and spatial frequency domain imaging (SFDI) [26] have been proposed. However, DRS and SFDI have insufficient spatial resolution to detect small tumors (< 1 mm^2^) and ESS was shown to have 69% sensitivity and 85% specificity [25]. Furthermore, of these imaging techniques, only light sheet microscopy using fluorescent labels [22] and SFDI [26] have been demonstrated on tissue areas approaching that of most BCS specimens (i.e. larger than 2 × 2 cm^2^). Extending these techniques to large breast tissue surfaces (e.g. 4 × 6 cm^2^) results in measurement times unacceptable for intra-operative use.

Raman spectroscopy is a highly sensitive optical technique that can provide a medical diagnosis based on quantitative molecular attributes of the tissue [27, 28]. Raman spectroscopy achieves molecular specificity by measuring the vibrational frequencies of tissue molecules excited by the laser. Basing the diagnosis on quantitative properties instead of human interpretation of structural images has been shown to reduce inter-observer variability [29]. Raman spectroscopy has been applied to the assessment of breast cancer with 94% sensitivity and 96% specificity [30], including hand-held fiber-probes for in vivo point-measurements [31, 32], albeit with no imaging capability and low spatial accuracy. Spatially offset Raman spectroscopy (SORS) was also proposed for tumors embedded within resected tissue [33, 34], but has thus far demonstrated only limited spatial resolution.

Spontaneous Raman spectroscopy alone is slow to image typical BCS specimens with sufficient spatial accuracy to allow accurate detection of small residual tumors that are of particular clinical interest. One approach for reducing the acquisition time is surface-enhanced Raman spectroscopy (SERS). A recent study by Wang et al. detected tumor at the excision surface with 89% sensitivity and 92% specificity by using gold nanoparticles functionalized with reporter SERS labels and monoclonal antibodies targeting biomarkers including epidermal growth factor receptor (EGFR), human epidermal growth factor receptor 2 (HER2), estrogen receptor (ER), or CD44 [35].

An alternative approach to reduce analysis time is selective-sampling Raman spectroscopy, which uses spatial information from the sample to guide Raman measurements [36–39]. This approach has the advantage that no exogenous labels are required. In a previous study, we have demonstrated the feasibility of multimodal spectral histopathology (MSH), a selective-sampling technique that combines high-resolution wide-field auto-fluorescence (AF) microscopy and Raman spectroscopy to detect ductal carcinomas in frozen breast micro-sections (5 × 5 mm^2^) [38]. This method acquires sensitive and specific Raman measurements while preserving the spatial resolution of AF images (10–20 μm), enabling MSH to identify small tumors. However, previous MSH studies were optimized for measurements of small tissue samples (less than 0.5 × 0.5 cm^2^) cut from surgical specimens. Extending these measurements to large breast tissue surfaces resulted in timescales unacceptable for intra-operative use (i.e. longer than 3 hours).

**Fig. 1:**
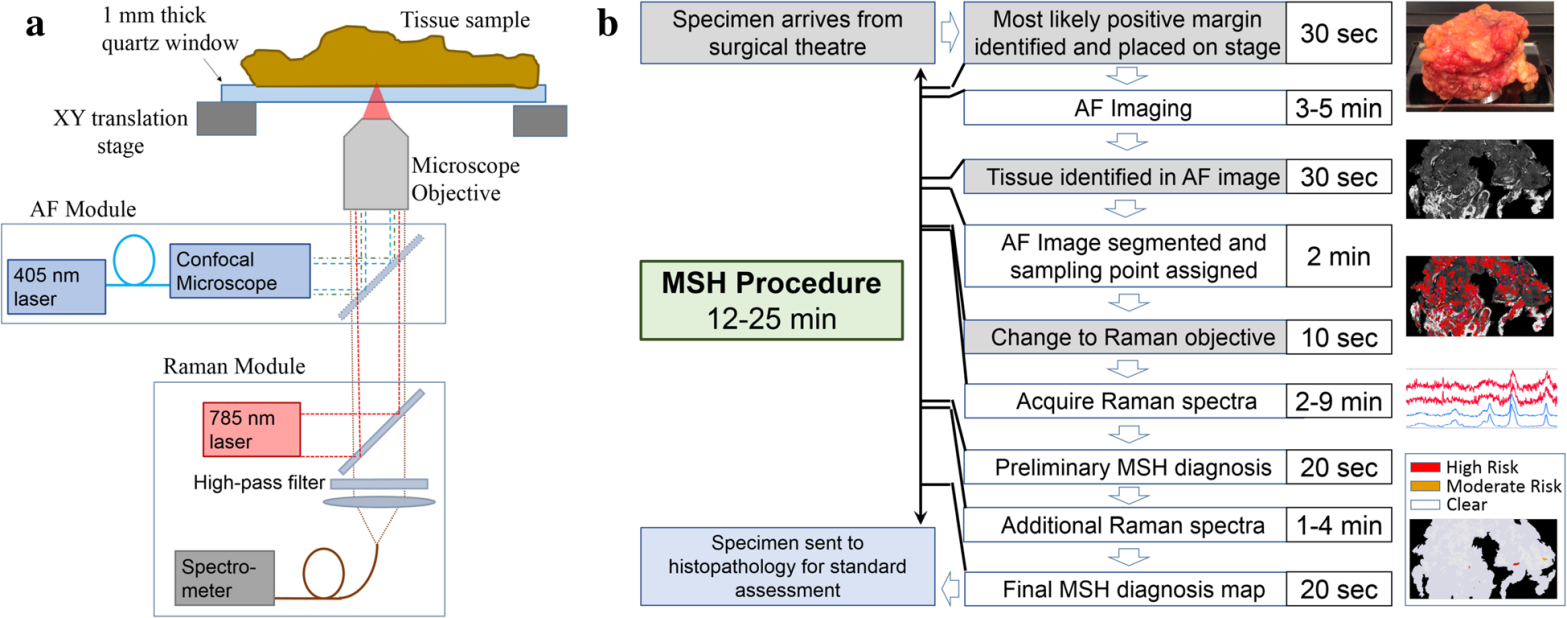
Instrument and procedure for multimodal spectral histopathology (MSH). **a** The MSH instrument consists of an inverted optical microscope with integrated Raman spectrometer (excitation 785 nm, detection Raman shift range 600–1800 cm^− **1**^) and confocal auto-fluorescence (AF) module (excitation 405 nm, detection range 450–520 nm). **b** The MSH measurement procedure can be completed in 12–24 min, depending on tissue size (up to 4 **×** 6.5 cm^**2**^). Steps in white boxes are automated (do not require user input). After MSH analysis, the tissue is returned for normal histopathology analysis

In this study, we have integrated an MSH instrument (combined confocal AF and Raman microscope, see Fig. 1a) and optimized the sampling and data processing algorithms combining spatial and spectral information for measuring the surface of large tissues (4 × 6.5 cm^2^ area) such as BCS specimens. The large-scale MSH technique reduces the number of required Raman measurements and allows analysis of the radial aspect of greatest concern in 12–24 min (see Fig. 1b), a timeframe considered appropriate for intra-operative use.

New data acquisition and analysis techniques were also automated to account for patient-to-patient variations and provide reliable, user-independent diagnosis across a broad range of tumor types and sizes. The segmentation and sampling algorithms were optimized to ensure consistent, thorough sampling of tumors on the surface of tissues with varying properties. The Raman spectral classifier was trained to distinguish between malignant and healthy tissues, even for difficult cases of hypercellular tissues. These spatial and molecular measurements were integrated into a final diagnosis image showing the presence or absence of tumor on the tissue surface. Through independent tests of these algorithms and measurement protocols on breast tissue samples and real, whole BCS specimen surfaces, we show that MSH has great potential for objective, intra-operative assessment of the excision surface of BCS specimens immediately after excision without requiring any sample preparation (sectioning or labeling).

## Methods

### Sample collection

Two sets of breast tissue samples were used in this study. Smaller samples of breast tissue cut from mastectomy samples were used for training (91 samples from 65 patients) and validation (70 samples from 56 patients) of MSH procedures and diagnosis algorithms. The mastectomy samples varied from 4 × 6 mm^2^ to 32 × 28 mm^2^ and were approximately 2–10 mm thick. These samples were frozen in liquid nitrogen and stored at − 20 °C until being thawed for measurement of one tissue surface. Principal component analysis (PCA) showed no differences between Raman spectra acquired from fresh and thawed samples. Samples known to contain confounding tissue types (e.g. fibroadenoma, fibrocystic change) were preferentially included in the training set to broaden the scope of the Raman spectroscopy classifier. After measurements, the samples were submitted for histological processing and hematoxylin and eosin (H&E) sections were obtained for each measured surface. Two validation samples in which conditions not included in the training set (e.g. metaplastic carcinoma) were discovered on histopathologic assessment were excluded from analysis. Future studies will target the inclusion of these rarer tissue types in the training set.

Fresh, uncut BCS specimens from 51 patients were measured as they arrived from the operating theater, without any preparation or processing. One surface was chosen for scanning based on proximity to palpable or visible lesions and to avoid high concentrations of surgical dyes. Following measurement, the scanned surface was colored with yellow ink and the specimen was evaluated by standard histopathological processes and protocols. This included cutting the specimen in a cruciate fashion and recovering H&E sections radially from the tumor to the tissue surface. Thus, these H&E sections were perpendicular to the surface measured by MSH. Pathologists reported the presence or absence of tumor on the measured surface marked in yellow.

### Multimodal MSH

A schematic of the MSH instrument and procedure is shown in Fig. 1. Tissues were placed directly onto a 5.1 × 7.6 cm^2^ quartz window for measurement in an inverted microscope configuration. Raman spectra were measured at the corners of the window and the window level was adjusted to reduce the tilt. Quartz is a popular substrate for Raman spectroscopy as it avoids fluorescence or scattering contributions common in other substrates. Tissues were found to be malleable such that their own weight pressed with sufficient force to ensure thorough, flat surface contact with the window.

AF images were acquired by a Nikon C2 confocal microscope module (405 nm laser, emission 511 nm long-pass filter, and detected by a photomultiplier tube). The portion of the AF image containing the tissue sample was automatically detected, allowing the background to be removed by a virtual mask. The user adjusted the intensity threshold for this algorithm by visual inspection to ensure appropriate masking. Dark regions or “segments” in the AF image were identified by an unsupervised algorithm. A threshold was varied automatically across several intensity values. For each threshold value, pixels with AF intensity values below the threshold were grouped into contiguous regions. Each contiguous region was marked as a segment. The threshold was varied to maximize the segmentation parameter *A·N*, where *A* is the area included in all segments within the image and *N* is the number of segments in the image. For MSH measurements, Raman measurement points were assigned within both dark segments and large regions of high AF intensity. The number and location of measurement points were determined as described in [40], with a minimum of two points per segment and a target density of one point per square millimeter.

Raman spectra were acquired by a fiber-coupled Raman spectroscopy module (785 nm excitation, 600–1800 cm^− 1^ detection). The procedure for Raman spectral acquisition differed depending on the phase of the study. In the initial training set measurement phase, regions of interest were identified by eye in the AF image. Raman spectra were then acquired in a raster scanning scheme. Scanned areas ranged from 3 × 4 to 20 × 16 mm^2^ with 40–100 μm step-sizes. For each scan, spectra were divided into four to eight groups based on spectral similarities by *k*-means cluster analysis. These groups were assigned arbitrary colors and the scan was displayed as a hyperspectral image. Under guidance of one or two trained pathologists, like-colored regions in the hyperspectral image were manually assigned to various tissue classes based on spatial correlation to AF and H&E images. Spectra within these regions were added to the training set for the corresponding tissue type. These tissue assignments are described in “Quantitative diagnosis based on Raman spectra”.

MSH-sampled measurements were generated from the raster scan using the nearest acquired spectra to the generated sampling points. These MSH measurements were limited to the raster-scanned area. For test set samples (both mastectomy tissue and whole BCS specimens), Raman measurement points were identified automatically by the segmentation and sampling procedure described above. For both of these schemes, the acquisition time was set to 0.3 s. All spectra were processed, analyzed, and classified individually.

Raman spectra were processed by standard algorithms including cosmic ray removal, wavenumber calibration, throughput correction, background subtraction [41], and smoothing [42]. Spectra with poor signal to noise ratio (SNR) were withheld from analysis (see Additional file 1 for details), removing approximately 2% of spectra from the training set. Raman raster scans from 91 breast tissue samples in the training set (28 with tumor, 63 without tumor, > 1000 spectra per sample, > 100,000 spectra total) were annotated and used to train a linear discriminant analysis (LDA) classification model as subsequently described.

Using this model, segments in the AF image were assigned class labels based on the Raman spectra acquired from the corresponding area. For samples included in the training set, spectral diagnoses were performed using a new classification model trained excluding the sample under evaluation (leave-one-out cross-validation). If the classification of spectra within a segment was not unanimous, the segment was split into smaller segments, each containing spectra with homogeneous diagnosis. Tissue regions diagnosed as tumor were assigned a second round of Raman measurements. Second round measurements were acquired with higher sampling density and doubled acquisition time per spectrum (0.6 s). In MSH measurements generated from raster scans, second round measurements averaged spectra from neighboring raster scan points.

First-round and second-round Raman spectra were used to create a final diagnosis image. For each segment, a tumor score (TS) ranging from 1 to 10 was calculated from the class probabilities returned by the LDA model for spectra within that segment. The TS for MSH measurements of training samples (see Additional file 1: Figures S3A and S4A) guided the creation of thresholds into “clear,” “moderate risk,” and “high risk” TS. These thresholds were applied to independent test MSH measurements of mastectomy samples and whole BCS excision surfaces to create three-color MSH diagnosis maps that could be quickly and easily interpreted in the operating theater.

### Statistical evaluation

The accuracy of the LDA classifier was estimated by fivefold cross-validation in which the spectra from 80% of patients (i.e. training set) were used to train a model to evaluate the remaining spectra (i.e. validation set). This was repeated five times to include each patient in the validation set once. Results were then reported on a per spectrum basis, including up to 1000 spectra per tissue type per sample.

For statistical evaluation of MSH diagnosis, a sample was considered positive if it contained tumor anywhere in the measured area. Likewise, the MSH diagnosis was considered positive if any tumor was identified (moderate risk or high risk) in the diagnosis image. Samples from mastectomy tissue were small enough that the MSH-identified tumor overlapped with histopathologically identified tumor in all cases where both were present. Similar correlation was not tested in BCS specimens as the H&E sections were obtained perpendicular to the MSH-measured surface per standard clinical procedure.

## Results

### Unsupervised segmentation of AF images

**Fig. 2:**
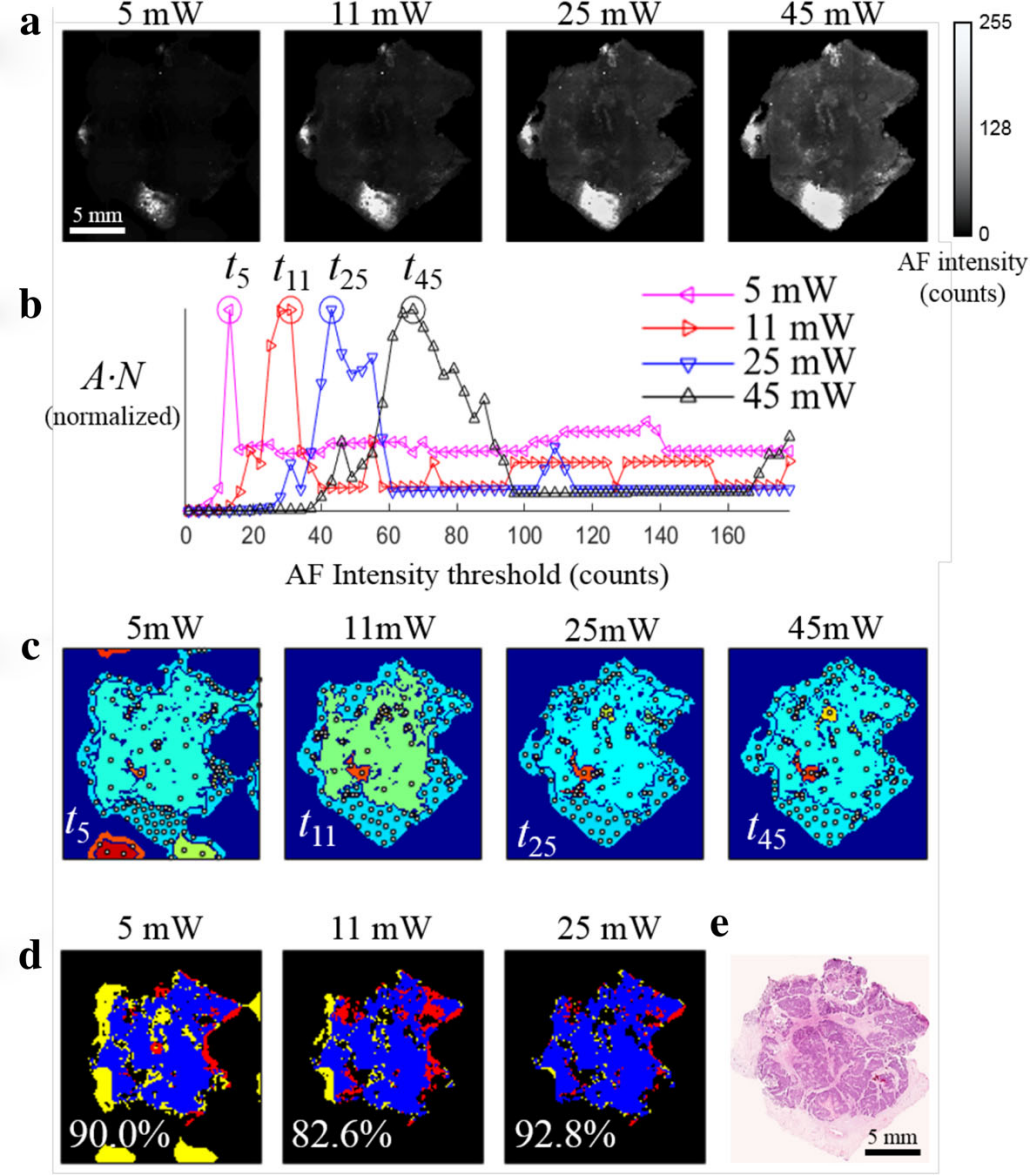
Method for unsupervised segmentation of auto-fluorescence (AF) images of breast tissue. **a** AF intensity images of a typical breast tissue sample containing invasive carcinoma obtained at difference excitation laser powers. **b** Representation of the total area captured by all segments to total number of segments for each image (*A·N*) versus the segmentation threshold. **c** Segmented AF images using the optimized intensity thresholds *t*_5_, *t*_11_, *t*_25_, *t*_45_; white dots indicate the sampling points for Raman spectroscopy. Each segment is assigned a unique, arbitrary color in these images. **d** The computed overlap with segmentation of the 45-mW image; blue, regions captured in segments in both AF images; red, regions were in segments in the 45-mW image but not in the images at lower power; yellow, regions in segments of AF image at lower laser power but not the 45-mW image. **e** Hematoxylin and eosin (H&E) section. The dense clusters of dark blue dots are tumor cells

Although the absolute origin of the signal in the AF signal is not fully understood, several endogenous fluorophores can be detected by the 405 nm excitation/511 nm long-pass emission system, including flavin adenine dinucleotide (FAD), reduced nicotinamide adenine dinucleotide (NADH), and collagen, the last of which is most common in stromal tissue [43]. In our observations within this study, dark regions in the acquired AF images generally contained tumor, benign growths (e.g. fibroadenoma), and adipose tissue (see Fig. 2). Therefore, these dark regions were identified as “segments” targeted for subsequent Raman measurements. The sampling algorithm also assigned additional Raman measurement points to bright regions in the AF image to address samples where the tumor may have higher AF intensity, i.e. samples containing only tumor and adipose tissues or with tumor cells scattered within stroma.

Automated algorithms were used to segment AF images and assign sampling points to minimize the number of Raman measurements while still acquiring spectra from any regions of tumor present on the surface of the sample. Each AF image was segmented by finding the maximum value of *A·N* (where *A* is the total area captured by all segments and *N* is the total number of segments) as a function of the segmentation intensity threshold. Maximizing *N* leads to the discrimination of small features while maximizing *A* favors larger segments, allowing faster measurements of large surfaces. The process of optimizing the segmentation and sampling algorithms toward these goals is described in “Optimization of segmentation and sampling algorithms” in Additional file 1.

The accuracy of the segmentation and sampling algorithms was ultimately evaluated by calculating the “tumor hit rate” as a figure of merit. The tumor hit rate describes the probability that a region of tumor on the surface of a sample will contain at least one Raman measurement. If the Raman spectral classifier were 100% accurate, the tumor hit rate would be equivalent to the sensitivity of the complete MSH procedure. The tumor hit rate was calculated for all 28 mastectomy samples containing tumor in the training set. For these samples, the median tumor hit rate was 100%. One sample contained approximately 5 mm^2^ of low-density tumor cells scattered within stroma that went unsampled. The tumor hit rates for the other mastectomy samples ranged from 73 to 100% (see Additional file 1: Figure S2). Therefore, this new method for optimizing the segmentation threshold allowed using fewer targeted Raman measurements to detect the majority of tumor regions over large surfaces. Indeed, the algorithm was optimized for assigning sampling points to large tissue areas detects most tumors - even those smaller than 1–2 mm - with a sampling density of one point per square millimeter. These algorithms allowed even large tissue surfaces (4 × 6.5 cm^2^) to be thoroughly analyzed by fewer than 2000 Raman measurements.

Sample to sample variations in the intensity of AF emission (depending on patient age, various tissue structures, etc.) is a key challenge when attempting to use an absolute intensity threshold for the segmentation of all AF intensity images. To ensure a user-independent and accurate diagnosis result, all data analysis steps were automated and designed to be invariant across the full range of samples. To evaluate the invariance of the segmentation algorithm to these conditions, we induced large AF intensity variations by imaging a set of eight breast tissue samples with four different excitation powers (5 mW, 12 mW, 25 mW, and 45 mW).

When the AF images recorded at different laser powers (Fig. 2a) were segmented using the intensity threshold values corresponding to the maximum values in Fig. 2b (*t*_5_, *t*_11_, *t*_25_, *t*_45_), consistent results were obtained regarding the shape and size of the dark segments and the generated locations of sampling points for Raman spectroscopy measurements (white dots) (see Fig. 2c). The percent overlap with the segments from the 45-mW image with AF images obtained at lower excitation powers ranged from 82 to 93%. Furthermore, segments identified in the image acquired at all laser powers correspond to the area of tissue containing tumor, shown by the dense clusters in the H&E image in Fig. 2e. These results indicate that the maximum value of the *A·N* function may provide a consistent, unsupervised, user-independent method for selecting an optimal intensity threshold for each AF image.

### Quantitative diagnosis based on Raman spectra

**Fig. 3:**
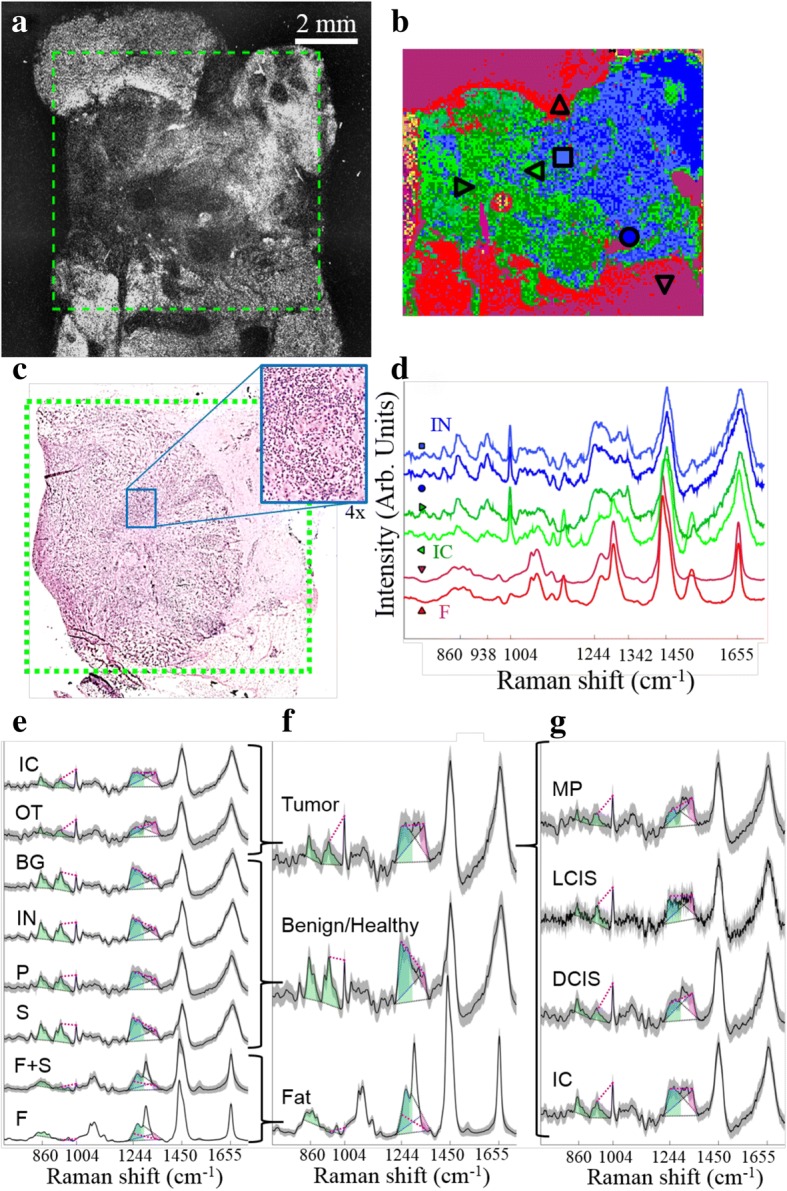
Raman spectral acquisition and annotation. Tumor regions (clusters of blue dots in the H&E image in (**c**)) appear darker in the auto-fluorescence (AF) image (**a**). The region in the green box was measured by a Raman raster scan. *K*-means cluster analysis of these spectra identifies similar spectra to create a hyperspectral image (**b**). Single spectra from locations marked in **b** are shown in **d**. Based on the information in **a-d**, pre-processed spectra from green areas (horizontal triangles) are marked as tumor, blue (square/circle) as inflamed stroma, and red (vertical triangles) as fat. Other clusters (cyan, yellow, and magenta) were background or noise and were withheld from the training set. Mean and standard deviation of all spectra in the training set show that the annotated tissue types (**e**) could be simplified to three classes used by the spectral classifier (**f**). Spectral features used for classification are marked as shaded areas (peak areas) and magenta lines (peak intensity differences). These peak areas are shaded blue for lipid-associated bands, green for protein-associated bands, and magenta for nucleic acid-associated bands. These features are consistent across all tumor types (**g**). Classes: IC, invasive carcinoma; OT, other tumor types (includes ductal carcinoma in situ (DCIS), lobular carcinoma in situ (LCIS), malignant phyllodes (MP)); BG, benign growths (includes fibroadenoma, sclerosing adenosis, hyperplasia); IN, inflammation; P, parenchyma; S, healthy stroma; F, fat; F + S, mixture of fat and stroma

After establishing a method for guiding Raman spectroscopy measurements based on AF images of breast tissue, a supervised model was developed for classification of breast tissues based on Raman spectra. Figure 3 compares the AF image (Fig. 3a), pseudo-color *k*-means clustering hyperspectral image from the Raman spectra (Fig. 3b), and the histopathology image obtained by H&E staining (Fig. 3c) for a typical breast sample containing invasive carcinoma, benign tissue with inflamed stroma, and fat. These images show that the *k-*means clustering images can accurately capture the main tissue structures, including the tumor, based solely on the molecular composition of tissue measured by the Raman spectra.

Under guidance of one or two trained breast pathologists, the *k*-means clustering hyperspectral images acquired from all mastectomy samples in the training set allowed for individual Raman spectra to be assigned a label corresponding to invasive carcinoma (IC), other tumor types (OT, e.g. DCIS, lobular carcinoma in situ (LCIS), malignant phyllodes (MP)), benign proliferative lesions (BG, e.g. fibroadenoma, sclerosing adenosis, epithelial hyperplasia), inflammation (IN), parenchyma (P), normal mammary stroma (S), fat (F), or a mixture of fat and stroma (F + S) (see Fig. 3e). A maximum of 1000 spectra of each tissue type was included from each sample. These eight tissue types were later relabeled into three classes based on spectral similarities: fat (including F and F + S), benign/healthy (including S, P, IN, and BG), and tumor (including invasive carcinoma (IC), DCIS, LCIS, and malignant phyllodes (MP)) (see Fig. 3f).

The simplified classes preserve major spectral features corresponding to cancer (nucleic acids, non-collagen proteins), stroma (collagen and other proteins), and fat (lipids) (see Fig. 3f) that are consistent with previously reported Raman spectra of breast cancers, adipose tissue, and other healthy breast tissue [27, 30, 38, 43]. Spectra from various tumor types (see Fig. 3g) share the characteristic features typical of tumor: intense bands assigned to nucleic acids (788 cm^− 1^, 1098 cm^− 1^, 1342 cm^− 1^), phenylalanine (1004 cm^− 1^), and amide I vibrations (1655 cm^− 1^), less intense bands corresponding to collagen (860 cm^− 1^, 938 cm^− 1^). Although there are spectral differences between these tumor types, they are less pronounced than the differences between spectra from tumor and other tissue types. These and other features (eight features in total) were identified to reduce the dimensionality of Raman spectra for more robust classification (see Additional file 1: Table S1). Briefly, spectra from tumor tissue was distinguished from benign tissues based on higher intensities in bands assigned to nucleic acids and lower intensities in collagen-assigned and amide III-assigned bands, in agreement with previous reports [38].

The model for classifying Raman spectra was optimized over several classifier families with varied parameters, including spectral features (see “Optimization of Raman Spectral Classifier” in Additional file 1). The overall sensitivity and specificity for the best-performing model (linear discriminant analysis (LDA)), were 90.2% and 93.4%, respectively (see Additional file 1: Table S3 for breakdown by tissue type). This represents the performance of the classifier on a single Raman spectrum, not taking into account any information from the AF image or neighboring spectra.

When the classifier performance was evaluated for different sub-types of tumor, the sensitivity was greater than 99% for DCIS, LCIS and malignant phyllodes, which were always found with closely packed tumor cells. However, the sensitivity was 89% for tissues containing invasive carcinoma, which often consisted of scattered tumor cells within benign tissue.

Another significant source of misclassification was spectra from benign/healthy tissues being classified as tumor. Although this “benign/healthy” class contains many tissue types such as stroma, parenchyma, and inflammation, classification errors occurred most often (50–80% specificity by spectrum) with spectra from hypercellular tissues including epithelial hyperplasia, sclerosing adenosis, and, to a lesser degree, fibroadenoma. Although these tissues were specifically targeted for inclusion in the training set, their low prevalence (three samples with sclerosing adenosis, four samples with hyperplasia, nine samples with fibroadenoma) suggest that the classifier could be further improved by including more measurements of these tissues in the training set.

### MSH tissue diagnostic model by integrating AF and Raman

The MSH diagnosis relied on both spatial information from segmented AF images and molecular information from Raman spectra. Within an AF image, the likelihood that a segment corresponded to a tumor (i.e. tumor score, TS) was calculated based on the Raman classification results of each spectrum within the segment.

**Fig. 4:**
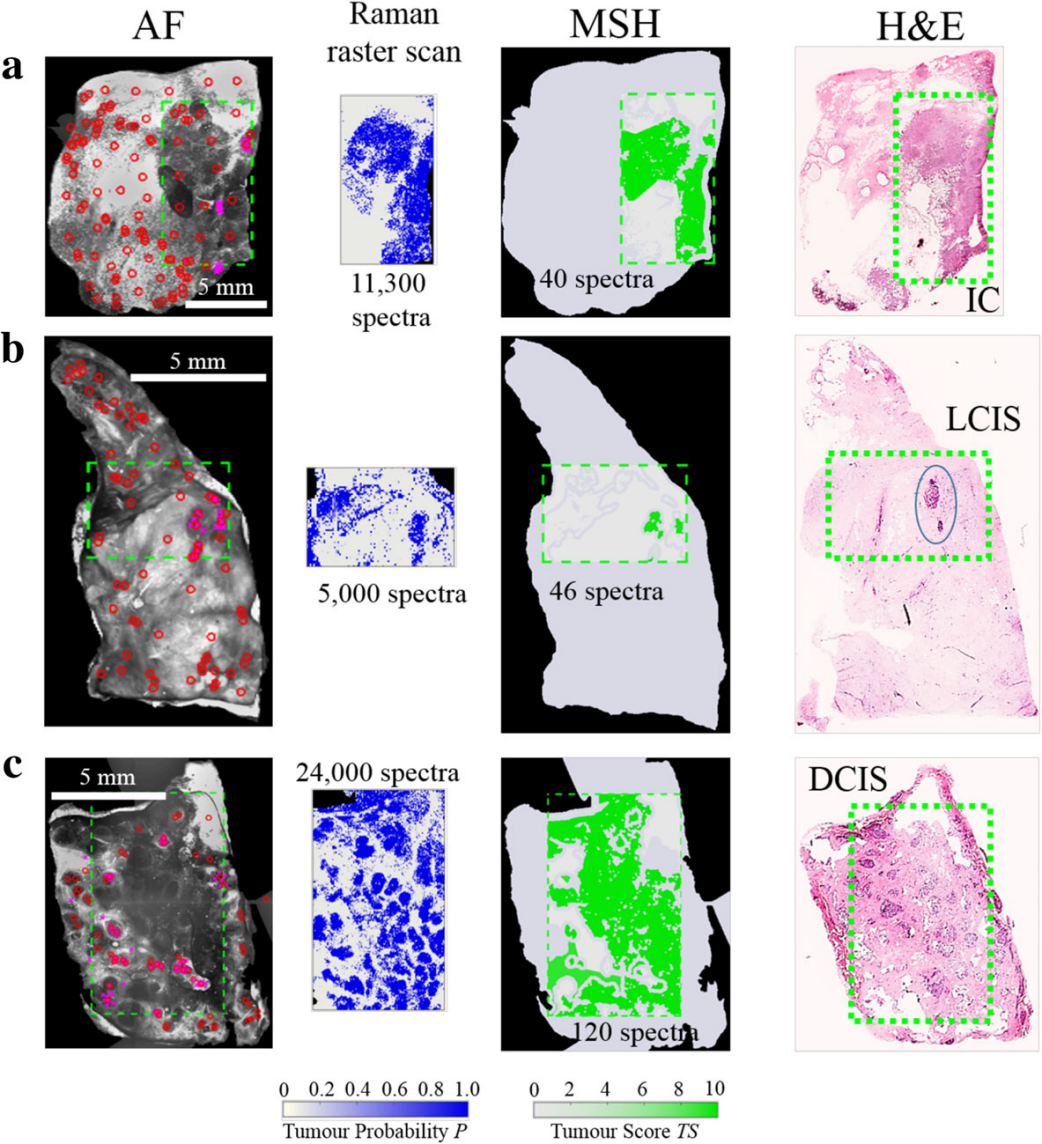
Multi-modal spectral histopathology (MSH) diagnosis generated using auto-fluorescence (AF) and raster scan Raman measurements of breast samples. Diagnosis for Raman raster scan is presented as tumor probability (*P*) (output of the classification model), while the diagnosis of each segment in the MSH is presented as tumor score (TS). Segmentation and sampling algorithms use AF images to focus Raman measurements (red circles) to suspicious regions, greatly reducing the number of spectra required for accurate diagnosis. Areas detected as tumor in the first round of MSH measurements are sampled by further Raman measurements (magenta crosses). **a**) Invasive carcinoma (IC); **b**) lobular carcinoma in situ (LCIS); **c**) ductal carcinoma in situ (DCIS)

To evaluate the performance of the MSH algorithm, we used leave-one-sample-out cross-validation to compare the MSH results for training set samples with the diagnoses obtained by raster-scanning Raman imaging and histopathology. An MSH diagnosis was obtained based on AF images and spectra from raster scan Raman measurements corresponding to locations assigned by the sampling algorithm. Figure 4 presents typical examples of MSH and Raman raster scan diagnoses for breast tissue samples containing the most common breast carcinomas: IC, LCIS, and DCIS. In all cases, the diagnostic images obtained by raster-scanning Raman spectroscopy and MSH were in agreement with histopathological H&E images. However, MSH dramatically reduced the acquisition time, as it required 100-fold to 200-fold fewer Raman spectra compared with raster scanning while providing similar diagnostic accuracy for breast carcinomas, even those comprising small tumors (< 1 mm^2^).

Under guidance from a trained breast cancer surgeon, the MSH diagnosis images were designed for ease of interpretation in the operating theater. The MSH results from training set samples were used to set thresholds to display the maps of TS as clear, moderate risk, or high risk. Setting the thresholds for the high-risk tumor at 9.9 (targeting high specificity) resulted in estimated sensitivity and specificity of 82% and 75%, respectively. The more sensitive moderate-risk threshold at 9.4 had estimated sensitivity and specificity for MSH of 96% and 59%, respectively.

### Independent test of MSH diagnosis on mastectomy samples

**Fig. 5:**
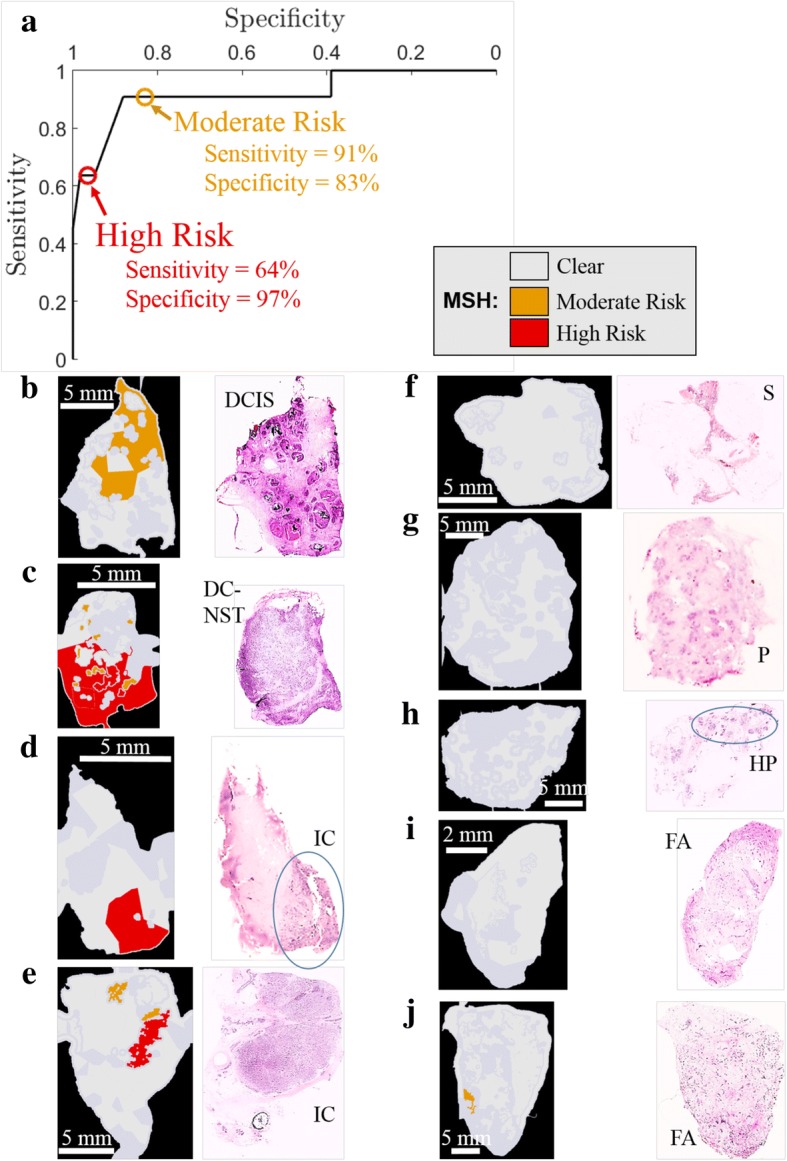
Validation of multi-modal spectral histopathology (MSH) on independent mastectomy breast samples. **a** Receiver-operator curve (ROC) for independent test samples at varying tumor score thresholds. Results corresponding to the thresholds determined based on training set data are marked with circles. (**b-e**) Examples of tumor tissue detected by MSH and confirmed by histopathology. DCIS, ductal carcinoma in situ; DC-NST, ductal carcinoma of no special type; IC, invasive carcinoma. **f-i** Examples of tissue identified as clear by both MSH and histopathology. S, stroma; P, parenchyma; HP, hyperplasia; FA, fibroadenoma. **j** Example of false positive where MSH marked segments as moderate risk although histopathological assessment identified fibroadenoma

The first independent validation of MSH was carried out on mastectomy samples (sizes ranging from 6 × 7 to 20 × 25 mm^2^) as H&E sections could be obtained from the measured surface to confirm the MSH diagnosis. Measurements for all tissue samples were performed according to the complete MSH protocol presented in Fig. 1b and lasted less than 4 min per sample. The receiver operating characteristic (ROC) curve for this test and examples of these MSH measurements are shown in Fig. 5. Tumor scores for all segments in independent mastectomy samples are shown in Additional file 1: Figure S4(B) with the maximum TS for each sample shown in Additional file 1: Figure S3(B).

Although a tumor such as DCIS may consist of many small tumor regions (0.2–1 mm), the main objective here was not to detect each individual microscopic region, rather to locate residual tumor within ~ 1 mm at the excision margin to facilitate intra-operative re-excisions. Thus, the sensitivity and specificity of the independent test samples was calculated by considering only the maximum TS found in the MSH measurement for the whole sample. Based on the moderate-risk TS threshold, the sensitivity and specificity were 91% and 83%, respectively. The results indicate successful detection of tumors, including DCIS consisting of tumor regions smaller than 1 × 1 mm^2^. Using the high-risk threshold increases the specificity to 97% while decreasing the sensitivity to 64%.

Thus, a surgeon observing a region diagnosed as high risk (see Fig. 5c-e) could remove more tissue from the corresponding region with high confidence of it being tumor. The surgeon would take action on a moderate-risk diagnosis (such as Fig. 5b) taking into account other information available at the time of surgery including patient history, disease type (e.g. DCIS), radiographic appearances, and size and location of detected tumor. The higher sensitivity of the moderate-risk threshold ensures that MSH misses few tumors on the excision surface.

### Proof of principle tests of MSH on whole BCS specimens in intra-operative timescales

Next, MSH measurements were acquired from 51 fresh, whole BCS specimens immediately after surgery with no sample preparation. The MSH measurements covered a surface area between 2 × 2 to 4 × 6.5 cm^2^ and were completed in 12–24 min. Simulating clinical application, a single side was analyzed that the surgeon may have considered of greatest concern.

**Fig. 6:**
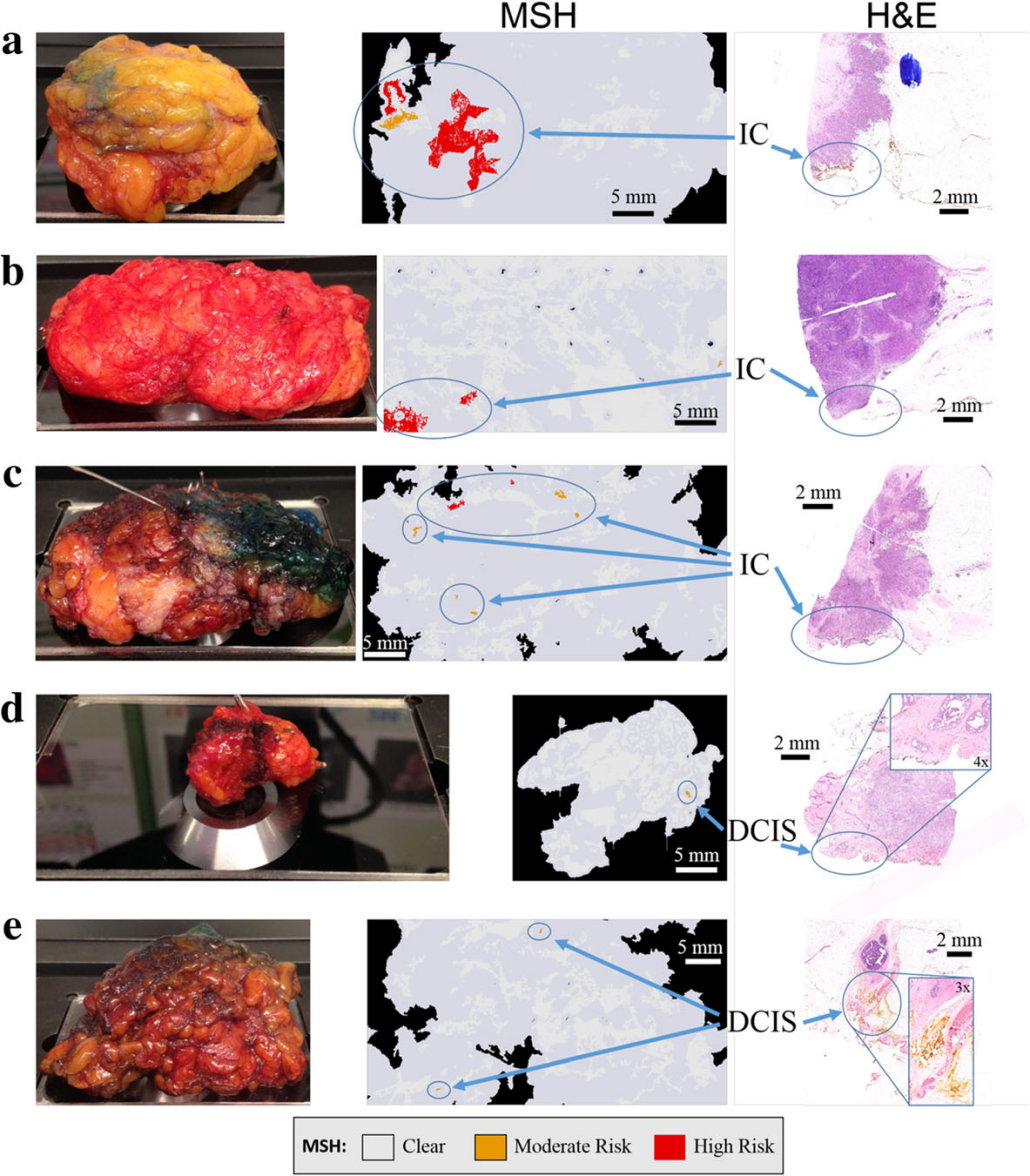
Examples of multi-modal spectral histopathology (MSH) measurements of whole breast conserving surgery (BCS) specimens with positive margins confirmed by histopathological assessment. The surface measured by MSH is facing downward in the specimen images. MSH detected tumor on the surface of all specimens in 12–24 min. **a**-**c**) invasive carcinoma (IC); **d**, **e**) ductal carcinoma in situ (DCIS)

MSH detected residual tumors on the surface of 18 BCS specimens. For 10 of the BCS specimens detected positive by MSH, the histopathological examination confirmed positive on-ink margins (see Fig. 6), including small (~ 1 × 1 mm^2^) pockets of DCIS (see Fig. 6d-e). MSH detected tumor in all specimens for which histopathological assessment identified positive margins (see all examples in Additional file 1: Figure S5).

**Fig. 7:**
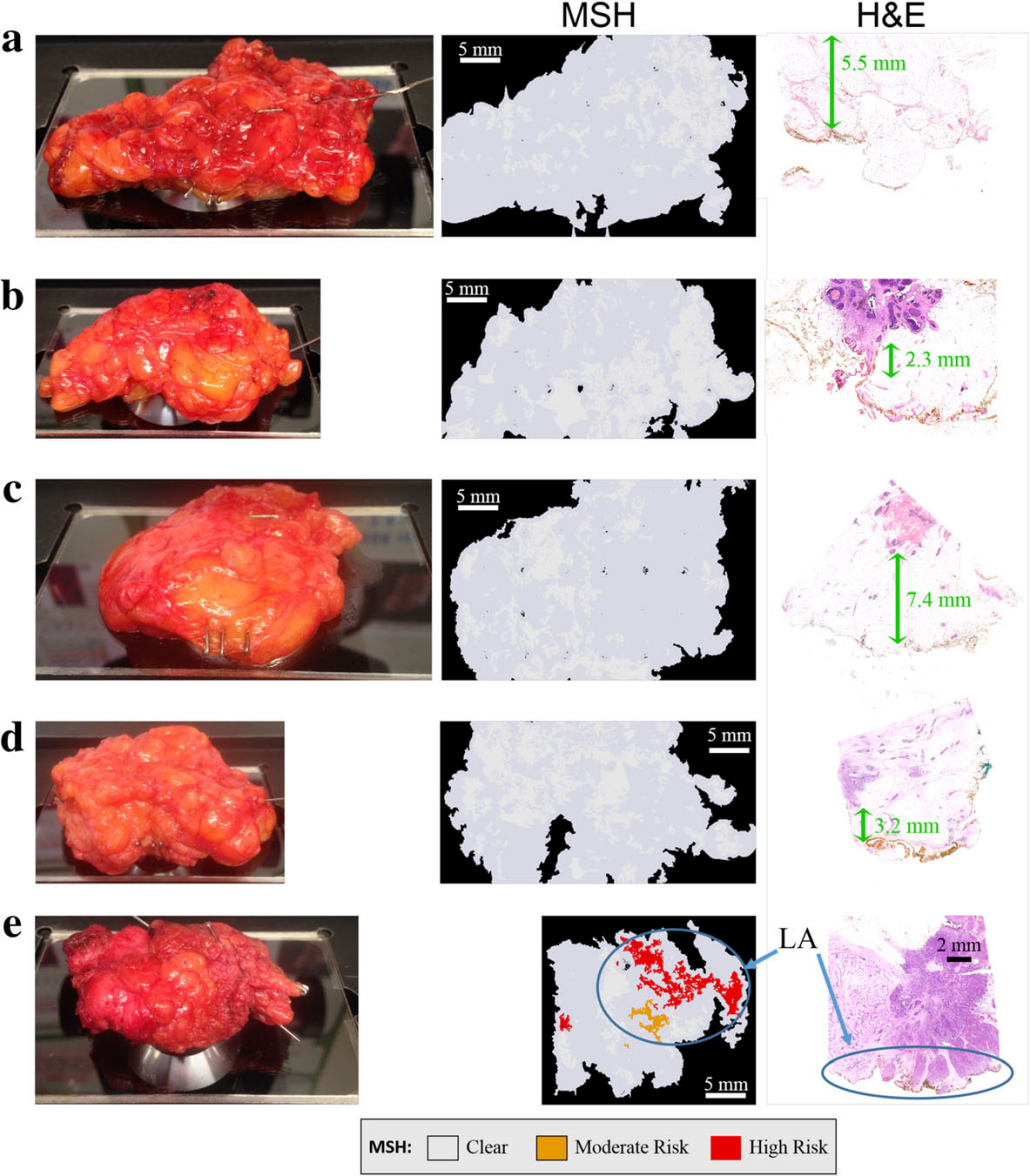
Examples of multi-modal spectral histopathology (MSH) measurements of whole breast conserving surgery (BCS) specimens for which histopathological examination identified negative margins. **a-d** MSH detected no tumor on the surface of 80% of specimens declared clear by histopathological examination. Distances from the measured margin to tumor are marked with green arrows. **e** MSH detected tumor although only lactation adenoma (LA) was found within 100 μm of the measured surface in sections sampled by histopathological examination

For eight specimens where MSH detected tumor, histopathological assessment identified margins wider than the penetration depth of our technique. Each of these measurements are presented in Additional file 1: Figure S6. Figure 7e shows an MSH measurement that detected positive margins. Histopathological assessment of this specimen identified lactation adenoma at the margin, but no tumor within 100 μm. For BCS specimens, histopathology sections were not available for the entire surface measured by MSH, only sections perpendicular to the measured surface. Therefore, these detected tumor regions could either be false positives or true positives not detected by histopathology. If these eight positive MSH results without confirmation by co-located histopathology sections are considered false positives, the specificity of MSH on BCS specimens is 80%, which is in agreement with the specificity for the independent test on mastectomy samples.

MSH provided a “clear” diagnosis in 33 BCS specimens (see Fig. 7a-d, all examples in Additional file 1: Figure S7). Normal histopathological assessment of these specimens detected no tumor near the measured surface. Therefore, MSH detected tumor on the surface of all specimens for which histopathological assessment later identified positive margins. For the 51 BCS surfaces measured in this independent test of MSH, the sensitivity was 100% and the specificity was at least 80%.

## Discussion

The main objective of this study was to evaluate the potential of multimodal spectral histopathology (MSH) to accurately detect tumors on the surface of excised BCS specimens within timescales compatible with intra-operative use. New measurement and data analysis algorithms were developed to obtain objective diagnoses of varied, large specimens free from user variability. These algorithms were optimized to measure a large tissue surface in intra-operative timescales while maintaining the ability to detect small tumors.

We aimed to assess the surface area of the specimen in the radial margin, which will enable assessment of one tissue surface plus approximately half of the adjacent surfaces at the same time due to fatty tissue deformation. Concentrating on the margins of greatest concern (surfaces up to 4 × 6.5 cm^2^) as informed by visual and tactile inspection of the specimen and intraoperative radiography could allow scanning in the time frame required for an intra-operative procedure (12–24 min).

Although DCIS occurs less often than IC, it often co-exists with small tumors and frequently extends beyond the boundaries of the index tumor, making re-excisions more common [5]. Small residual foci of DCIS are difficult to detect by alternative intra-operative techniques under development because of limited spatial resolution or sampling coverage. However, MSH utilizes the high spatial resolution, speed, and sensitivity (but low specificity) of AF imaging to guide Raman spectroscopy with its high chemical specificity to detect small tumors. Indeed, MSH was able to detect residual DCIS and other small tumors (1–2 mm) on the surface of whole BCS specimens that were missed during surgery.

Mastectomy samples were chosen for developing the diagnosis model and the initial independent test because H&E sections could be obtained from the same surface measured by MSH, thus providing a reliable standard of reference. These tests estimated the sensitivity and specificity of the technique as 91% and 83%, respectively. These results included challenging cases (highly proliferative but non-malignant lesions) that were under-represented in the training set for the classification model. Still, MSH provided accurate detection of breast carcinomas including DCIS.

The validation of the technique using 51 whole BCS specimen surfaces (4 × 6.5 cm^2^) measured immediately after surgery allowed demonstration of the feasibility of intra-operative use of MSH (12–24 min). MSH detected tumors in all scanned surfaces that had positive margins subsequently confirmed by histopathological assessment, including those with DCIS. Had the MSH results been available in the operating theater, the residual tumor may have been immediately removed.

As standard histopathology practice sparsely involves sectioning BCS tissue perpendicular to the surface measured by MSH, H&E sections were not available at all locations where MSH detected tumor. Although MSH provides a more comprehensive analysis of the excision surface compared to slide-based histology, histopathology obtains information such as tumor type and progression, which is important for continuing patient care, but not urgently required during surgery. The non-destructive, non-labeling nature of MSH allows BCS specimens to be submitted for normal histopathological processing following the MSH measurement.

These results suggest that clinical use of MSH could detect 95% of residual tumors in BCS surgeries and prevent re-excisions in these cases. Positive margins remaining undetected by MSH would proceed through treatment following current protocols. Any false positives would result in cavity shaves, similar to the untargeted approach of Chagpar et al. [6]. Thus, MSH can be used with minimal risk and great potential benefit to the patient.

Our investigation confirms the extension of the MSH technique to real specimens. The quartz window (5.1 × 7.6 cm^2^) was able to accommodate most BCS specimens. Within 12–24 min, the excision surface of greatest concern could be measured with diagnostic results displayed as three-color images, allowing surgeons to make immediate, informed decisions on further resections while incorporating additional clinical factors. Nevertheless, the analysis time can be further reduced in the future by developing a more optimized and automated instrument to eliminate the current manual steps (e.g. microscope focusing, change between AF and Raman objectives, and faster microscope translation stage [39]). In clinical use, additional information such as radiographic images of the specimens would be used to identify the surface with the highest risk, allowing prioritization of faster or more accurate measurements (e.g. by increasing the Raman acquisition time over a smaller area). Faster multi-beam Raman spectroscopy could also be used to parallelize the acquisition of the Raman spectra of tissue [44] to provide additional speed and allow the measurement of the entire specimen surface within a shorter measurement time. With such further development and integration into clinical practice, many re-excision operations may be prevented.

## Conclusion

Combining the fast, high-resolution imaging of AF and the accurate molecular diagnosis of Raman spectroscopy, MSH is able to identify small residual tumors on the surface of breast excision specimens within intra-operative timescales. Measurement and diagnosis algorithms have been trained and optimized to quickly evaluate large tissue surfaces. A future fully automated system will further improve on accuracy and speed. MSH diagnosis images could guide the surgeon to remove additional tissue immediately and potentially prevent a large number of secondary operations.

## Additional file

Additional file 1: **Figure S1.** Detailed performance of the linear discriminant analysis (LDA) Raman spectral classifier. **Figure S2.** Optimization of auto-fluorescence (AF) segmentation (A-E) and sampling (F) algorithms. **Table S1.** Molecular assignments of Raman spectral features and relative prevalence in each tissue class. **Table S2.** Performance of various classification models on Raman spectra from breast samples in the training set. **Table S3.** Confusion matrix for Raman spectral classifier based on fivefold cross-validation for breast samples in the training set. Statistics are median values for 10 re-partitions of fivefold cross-validation. **Figure S3.** Histogram of maximum tumor scores for each sample in the training set (A) and independent test set (B). Samples containing a segment with a tumor score greater than 9.0 were considered positive in this study. **Figure S4.** Tumor scores for all segments in mastectomy tissue samples from the training set (A) and independent test set (B). **Figure S5.** All BCS specimens with positive margins detected by MSH and confirmed by histopathology. **Figure S6.** All BCS specimens for which MSH detected positive margins but no tumor was found at the surface in sections sampled by histopathology. **Figure S7.** All BCS specimens for which the measured surface was diagnosed as clear by both MSH and histopathology. (DOCX 5487 kb)

## Abbreviations

AF: Auto-fluorescence
BCS: Breast conserving surgery
DCIS: Ductal carcinoma in situ
DRS: Diffuse reflectance spectroscopy
ESS: Elastic scattering spectroscopy
FAD: Flavin adenine dinucleotide
FLIm: Fluorescence lifetime imaging
H&E: Hematoxylin and eosin
IC: Invasive carcinoma
LCIS: Lobular carcinoma in situ
LDA: Linear discriminant analysis
MSH: Multi-modal spectral histopathology
NADH: Reduced nicotinamide adenine dinucleotide
PCA: Principal component analysis
ROC: Receiver operating characteristic
SERS: Surface-enhanced Raman spectroscopy
SFDI: Spatial frequency domain imaging
SORS: Spatially offset Raman spectroscopy
TS: Tumor score
