# Supplementary material for: Intra-operative spectroscopic assessment of surgical margins during breast conserving surgery
Source: Breast Cancer Res. 2018 Jul 9;20:69. doi: 10.1186/s13058-018-1002-2 (PMC6038277; doi:10.1186/s13058-018-1002-2)
Supplement: Supplementary file 1 — Figure S1. Detailed performance of the linear discriminant analysis (LDA) Raman spectral classifier. Figure S2. Optimization of auto-fluorescence (AF) segmentation (A-E) and sampling (F) algorithms. Table S1. Molecular assignments of Raman spectral features and relative prevalence in each tissue class. Table S2. Performance of various classification models on Raman spectra from breast samples in the training set. Table S3. Confusion matrix for Raman spectral classifier based on fivefold cross-validation for breast samples in the training set. Statistics are median values for 10 re-partitions of fivefold cross-validation. Figure S3. Histogram of maximum tumor scores for each sample in the training set (A) and independent test set (B). Samples containing a segment with a tumor score greater than 9.0 were considered positive in this study. Figure S4. Tumor scores for all segments in mastectomy tissue samples from the training set (A) and independent test set (B). Figure S5. All BCS specimens with positive margins detected by MSH and confirmed by histopathology. Figure S6. All BCS specimens for which MSH detected positive margins but no tumor was found at the surface in sections sampled by histopathology. Figure S7. All BCS specimens for which the measured surface was diagnosed as clear by both MSH and histopathology. (DOCX 5487 kb) [file 13058_2018_1002_MOESM1_ESM.docx]

Intra-operative spectroscopic assessment of surgical margins
during breast conserving surgery

**Additional file 1**

*Choice of sample sizes*

The study was designed to determine the optimal measurement technique combining information from confocal auto-fluorescence (AF) and Raman spectroscopy to evaluate the presence of tumor at the surface, or “on-ink” margin, of BCS specimens. As no previous information was available regarding the effect size, the size of the training set was not set at the start. Instead, the performance of the classification model was periodically evaluated by cross-validation as a function of the number of patients, and the decision to stop was made when a saturation of classification was observed (*i.e.* very little improvement by including more samples) (see Figure S1(A)). The results using 91 samples (28 containing tumor) from 65 patients showed that Raman classification models indicated saturation once the training set contained approximately 50 patients. Therefore, these 91 samples were used to train the Raman classifier and optimize the AF algorithms. The observed saturation at approximately 50 patients also provided a target number of patients to include in independent test MSH measurements of mastectomy tissue (72 samples, 13 containing tumor, from 57 patients - no overlap with training set samples) and whole BCS specimens (51 samples from 51 patients). Within the practical and ethical constraints of this observational study, all available tissues were included.


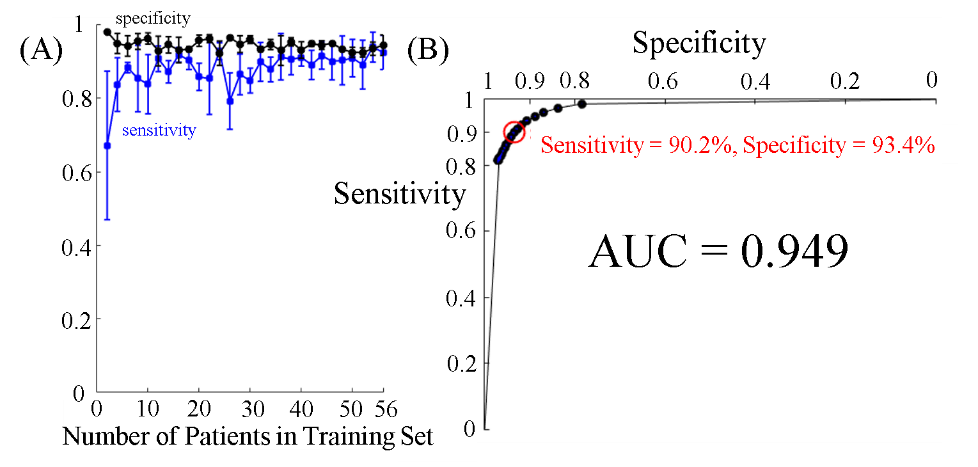


Figure S1: Detailed performance of the linear discriminant analysis (LDA) Raman spectral classifier. (A) The model becomes more accurate with a larger training set before saturation. (B) The receiver-operator curve (ROC) for the LDA model (based on five-fold cross validation) shows the flexibility of the classifier by varying the class probability threshold for classification.

*Instrument and procedure for confocal auto-fluorescence*

Auto-fluorescence (AF) images were obtained by scanning across the surface of the tissue through a 2x, 0.1 NA objective (Plan Apo, Nikon), and the fluorescence light was collected and passed through a 511 nm longpass filter (FF511-Di01, Chroma) and detected by the photomultiplier tube (PMT) included in the Nikon C2 confocal attachment. The field of view of the confocal attachment and the 2x objective was 6.48 x 6.47 mm^2^. Samples larger than this were measured by acquiring and stitching together tiled images. Individual tiles were flattened by fitting vertically and horizontally averaged intensity values with second order polynomials during the stitching process.

*Optimization of Segmentation and Sampling Algorithms*

During the segmentation algorithm, the segmentation parameter is calculated for each intensity threshold. A number of segmentation parameters were evaluated, including *N*, $A\cdot N^{2}$, $A\cdot N$, and $A\sqrt{N}$, where *N* is the number of segments and *A* is the total area of all segments. Each of these parameters was applied to AF images of training set samples containing tumor. Based on the analysis described below, $A\cdot N$ was chosen as the optimal segmentation parameter.

The effectiveness of the segmentation algorithm was evaluated in part by measuring the percentage of tumor regions captured in the segments. We named this metric “segment overlap,” as it represents the likelihood that a tumor will be overlapped by a segment. All 28 samples containing tumor in the training set were used to optimize segmentation algorithms (see Figure S2). Using the optimized segmentation algorithm based on $A\cdot N$, the segment overlap for these samples ranged from 8% to 78% with a median value of 40%. In all samples, some portion of the tumor was contained in at least one segment. As the aim of the technique is to inform the surgeon whether any residual tumor is detected at the resection surface (*i.e.* yes or no) with a spatial accuracy of approximately 1 mm, the overlap with tumor in every sample and overall 40% segment overlap was considered acceptable.

For all segments in an AF image containing tumor, we also calculated the percentage of tumor area. This metric, named “segment tumor percentage,” is related to the likelihood that sampling points within these segments will hit the tumor. Taking the segment with maximum value from each training sample, the segment tumor percentages ranged from 5% to 100% with a median value of 100% (Figure S2(C)). This implies that in the majority of the training samples containing tumor, at least one segment contains only tumor tissue. Including all segments containing tumor in the training samples (Figure S2(B)), the median segment tumor percentage was 52%, with a range of 5% to 99%.

Some samples contained only adipose and tumor tissue. Other samples contained tumor cells scattered throughout stromal tissue. In these cases, the regions with tumor have *higher* intensity in the AF images. To guard against these situations, the segmentation algorithm marks large regions of the remaining tissue area as additional segments. Following the first step of segmentation, additional segments are marked in large areas of the remaining surface. These additional segments are constructed by trimming the remaining area until small regions disappear. The amount of trimming was optimized on training set AF images using the performance measures segment overlap and segment tumor percentage.


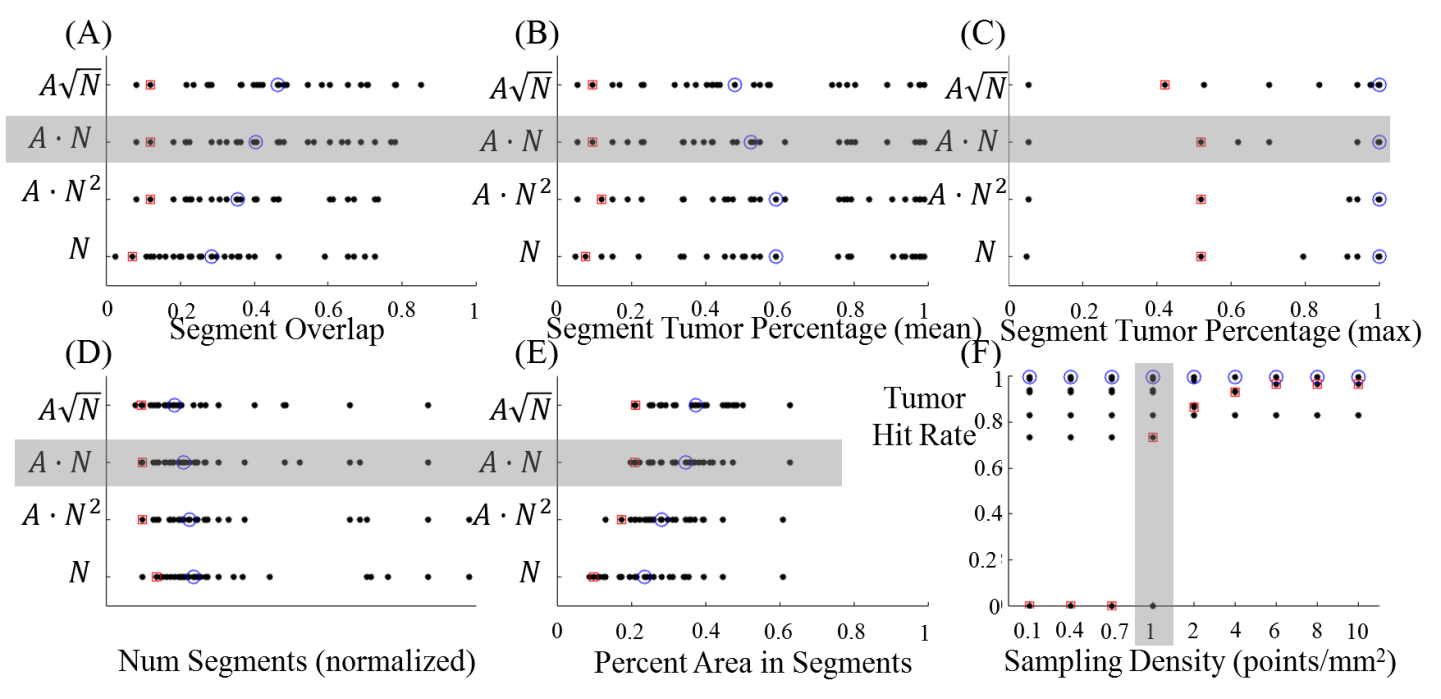


Figure S2: Optimization of auto-fluorescence (AF) segmentation (A-E) and sampling (F) algorithms. Blue circles mark median values and red squares mark 5^th^ percentile values. As the segmentation parameter is changed to favor larger contributions from the segmented area *A* compared to the total number of segments *N*, segmentation solutions are chosen with less segments (D) and a larger percentage of area in segments (E). The larger segments cause the segment overlap, *i.e.* the percentage of tumor area contained in a segment, to increase (A) and the segment tumor percentage, *i.e.* the percentage of tumor area contained in a segment, to decrease (B and C). The segment tumor percentage is related to the likely hood that the tumor will be sampled within the segment. The optimal segmentation parameter was chosen to be *A·N* (shaded) based on the improvement in segment overlap without sacrificing a large degree of segment tumor percentage. (F) The optimal sampling density for Raman measurements was chosen based on the Hit Rate, *i.e.* the percentage of tumor area that has been sampled. The optimal sampling density of 1 point/mm^2^ (shaded) corresponds to 0.8 minute average measurement time. Denser sampling resulted in slight improvements in Hit Rate but longer Raman measurement times (*e.g.* 1.3 minute average for 2 points/mm^2^).

The above statistics do not include segments assigned to the remaining large bright regions after initial segmentation that are typically assigned to stroma. However, the segmentation algorithm must also automatically function for AF images of samples containing tumor only, or tumor and fat with no stroma, or tumor cells scattered within bright stroma areas. When segments corresponding to such samples were included in the analysis, we found the minimum, maximum, and median segment tumor percentages (maximum for each sample) increased to 22%, 83%, and 61%, respectively. The minimum, maximum, and median segment tumor percentages when including all tumor-containing segments were 5%, 99%, and 47%, respectively.

Parameters of the segmentation algorithm, including the segmentation parameter (*e.g.* $A\cdot N$) and bright segment trimming size, were optimized to maximize the segment overlap (Figure S2(A)) while also maximizing the segment tumor percentage (Figure S2(B-C)), ensuring thorough sampling of surface regions containing tumor. These were simultaneously balanced against the total number of segments and percentage of area contained in segments (Figures S2(D) and (E), respectively) to minimize the required number of Raman measurements and thereby reduce the total measurement time.

The algorithm describing the assignment of Raman sampling points within segments has been described previously [40]. Briefly, a minimum of two sampling points are assigned to each segment. Additional sampling points are assigned to reach an average sampling density of one measurement per square millimeter. Preference for these additional points is given to segments with more variation of AF intensity values.

The sampling density was also optimized on training set AF images. The performance of various sampling densities was compared based on the total number of sampling points (which determines the Raman measurement time) and the “Tumor Hit Rate,” which is the percentage of tumor area containing at least one sampling point. These results are shown in Figure S2(F). Based on this analysis, the sampling density was set at 1 point per mm^2^ (corresponding to 0.8 minute median Raman measurement time). Higher sampling densities achieved higher Tumor Hit Rates, but at the expense of longer – and, in the authors’ view, impractical – measurement times.

While the segment overlap and segment tumor percentage are useful in optimizing the segmentation algorithms, the ultimate performance metric of the algorithm is determined by the Tumor Hit Rate. The Tumor Hit Rate would be equivalent to the overall sensitivity of MSH for a perfectly accurate Raman spectroscopy classifier (100% sensitivity and 100% specificity). For the 28 breast samples in the training set containing tumor, the Tumor Hit Rate ranged from 0% (one sample, second lowest was 73%) to 100% with a median of 100%.

*Instrumentation for Raman spectroscopy*

Excitation for Raman spectroscopy is driven by a 785 nm CW laser (XTRA, Toptica). This laser is focused on the sample by a 60x, 0.85 NA objective optimized for Raman spectroscopy at 785 nm excitation (River Diagnostics). The power at the sample was 150 mW. No tissue damage was observed due to AF or Raman laser illumination. Scattered light was collected by the same objective and delivered through a dichroic mirror and longpass filter (RT785rdc and RET792lp, Chroma) and focused by a 25 mm focal length lens (AC127-025-B-ML, Thorlabs) to a 100 µm core optical fiber. The fiber delivered this light to a spectrometer (Oriel 77 200, Newport) and charge coupled device (CCD) camera (iDus DU-401-A-RR-DD, Andor). Spectra were detected over the range of 600‑1800 cm^-1^.

The signal-to-noise ratio (SNR) for each spectrum was calculated as the intensity of the 1450 cm^‑1^ CH_2_ scissors peak divided by the standard deviation of intensities in the relatively quiet range 1370-1410 cm^‑1^. Spectra with SNR less than 20 were withheld from analysis. Approximately 98% of all spectra included in the training set had SNR above this threshold. Throughput correction was performed using a NIST fluorescence standard (SRM 2241, NIST).

*Optimization of Raman Spectral Classifier*

All pre-processing and spectral feature parameters (including model type, class probability threshold, etc.) were selected based on the five-fold cross-validation classification performance on the training set. Optimization was partially carried out using the University of Nottingham High Performance Computing facility. Patients in the training set were randomly divided into five groups. Raman spectra from four of these groups were used to train the classifier. The remaining group was used as a validation set to measure the classifier performance. This process was repeated using each of the five groups as the validation set.

Dimensionality reduction techniques (feature extraction, principal component analysis (PCA)) were used with various classification models including linear discriminant analysis (LDA), quadratic discriminant analysis (QDA), artificial neural network (ANN), multinomial logistic regression (MNLR), k-nearest neighbor (KNN), and random forest (RF). Each of these models was optimized by varying the number and combination of spectral features (or number of principal components (PCs) as well as other parameters specific to each model. For models that calculated class probabilities (LDA, QDA, ANN, MNLR), the sensitivity and specificity of these models could be varied by setting a threshold of the calculated class probability corresponding to tumor. For example, with the threshold set at the 92^nd^ percentile of tumor probability, any spectrum with a tumor probability above this threshold were classified as tumor, giving the model a 92% sensitivity on the training set. For each of these models, the optimal classifier performance was achieved with the training set sensitivity set to 92%. Out of the 46 spectral features analyzed, eight were included in the final model. The molecular assignments for these features are described in Table S1 [27].

| Feature Location (cm^‑1^) | Assignment | Prevalence in Fat | Prevalence in Benign | Prevalence in Tumor |
| --- | --- | --- | --- | --- |
| A_860_ | collagen | 0 | ++ | + |
| A_938_ | C-C collagen | 0 | ++ | + |
| A_1244_ | Amide III | 0 | ++ | + |
| A_1250_ | combination of Amide III peaks | + | ++ | + |
| A_1264_ | =CH | ++ | ++ | + |
| A_1342_ | adenine | 0 | 0 | ++ |
| I_1004_ - I_938_ | 1004 = phenylalanine ring breathing, 938 = collagen | + | 0 | ++ |
| I_1342_ - I_1244_ | 1342 = adenine, 1244 = Amide III | 0 | - | + |

Table S1: Molecular assignments of Raman spectral features and relative prevalence in each tissue class. Spectra from tumor are identified by increased concentration of nucleic acids and a lower ratio of collagen to other proteins. A = integrated band area, I = peak intensity value.

The results of the various spectral classifiers are shown in Table S2. Based on this analysis, an LDA model with eight spectral features was chosen for training the final Raman classification model. Further details of the performance of this LDA model are shown in Table S3 and Figure S1. Figure S1(A) shows the improvement of the model as more patients are included in the training set until eventual saturation. Figure S1(B) shows the receiver-operator curve (ROC) as the training set sensitivity is varied. Movement along this curve allows the sensitivity of the model to be adjusted based on the calculated class probabilities.

| Model Type | Details | Sensitivity | Specificity |
| --- | --- | --- | --- |
| Linear Discriminant Analysis (LDA) | 8 features | 90.2% | 93.4% |
| Quadratic Discriminant Analysis (QDA) | 6 features | 90.2% | 92.5% |
| Artificial Neural Network (ANN) | 8 features; 5 neurons in hidden layer | 89.1% | 93.7% |
| ANN – 2 layers | 8 features; 4, 3 neurons in each hidden layer | 89.2% | 93.6% |
| Multinomial Logistic Regression (MNLR) | 10 features | 89.5% | 93.8% |
| K-Nearest Neighbors (KNN) | 8 features | 62.2% | 96.9% |
| Random Forest (RF) | 8 features; 10 trees; 200 minimum leaf size | 76.4% | 97.6% |
| Principal Component Analysis (PCA) – LDA | 4 principal components (PCs) | 88.9% | 94.2% |
| PCA – ANN | 4 PCs; 5 neurons in hidden layer | 87.2% | 94.4% |

Table S2: Performance of various classification models on Raman spectra from breast samples in the training set. Each of these models was optimized by adjusting the included spectral features and other parameters. Linear discriminant analysis (LDA) showed the best performance and was chosen for application.

| Known\Predicted | Fat | Benign/Healthy | Tumor |
| --- | --- | --- | --- |
| Fat | **97.1%** | 0.6% | 2.3% |
| Benign/Healthy | 2.3% | **83.1%** | 14.5% |
| Tumor | 0.8% | 8.9% | **90.2%** |
| Invasive carcinoma  (26 patients) | 0.9% | 10.1% | **89.0%** |
| Ductal carcinoma *in situ* (DCIS)  (4 patients) | 0.5% | 0.2% | **99.4%** |
| Lobular carcinoma *in situ* (LCIS)  (1 patient) | 0.0% | 0.5% | **99.5%** |
| Malignant phyllodes  (1 patient) | 0.7% | 0.0% | **99.3%** |

Table S3: Confusion matrix for Raman spectral classifier based on five-fold cross-validation for breast samples in the training set. Statistics are median values for ten re-partitions of five-fold cross validation.

*Calculation and Thresholding of Tumor Score (TS)*

To obtain a diagnosis for each segment incorporating all Raman spectra acquired within that segment, the classification results from these spectra were combined into a single number. The Tumor Score (*TS*) is a metric ranging from 1-10 calculated as $TS={10}^{[1-\prod_{i} \left( 1-P_{i} \right)]}$ from the tumor probability, *P_i_,* estimated by the Raman spectral classifier for each spectrum within the segment. *P_i_* is capped to a maximum value of 0.9 to limit the diagnostic influence of a single spectrum. Figure S4(A) shows the Tumor Scores for MSH measurements of all training samples. These Tumor Scores guided the creation of thresholds into “Clear,” “Moderate Risk,” and “High Risk” categories for simplified visualization of MSH diagnosis images.


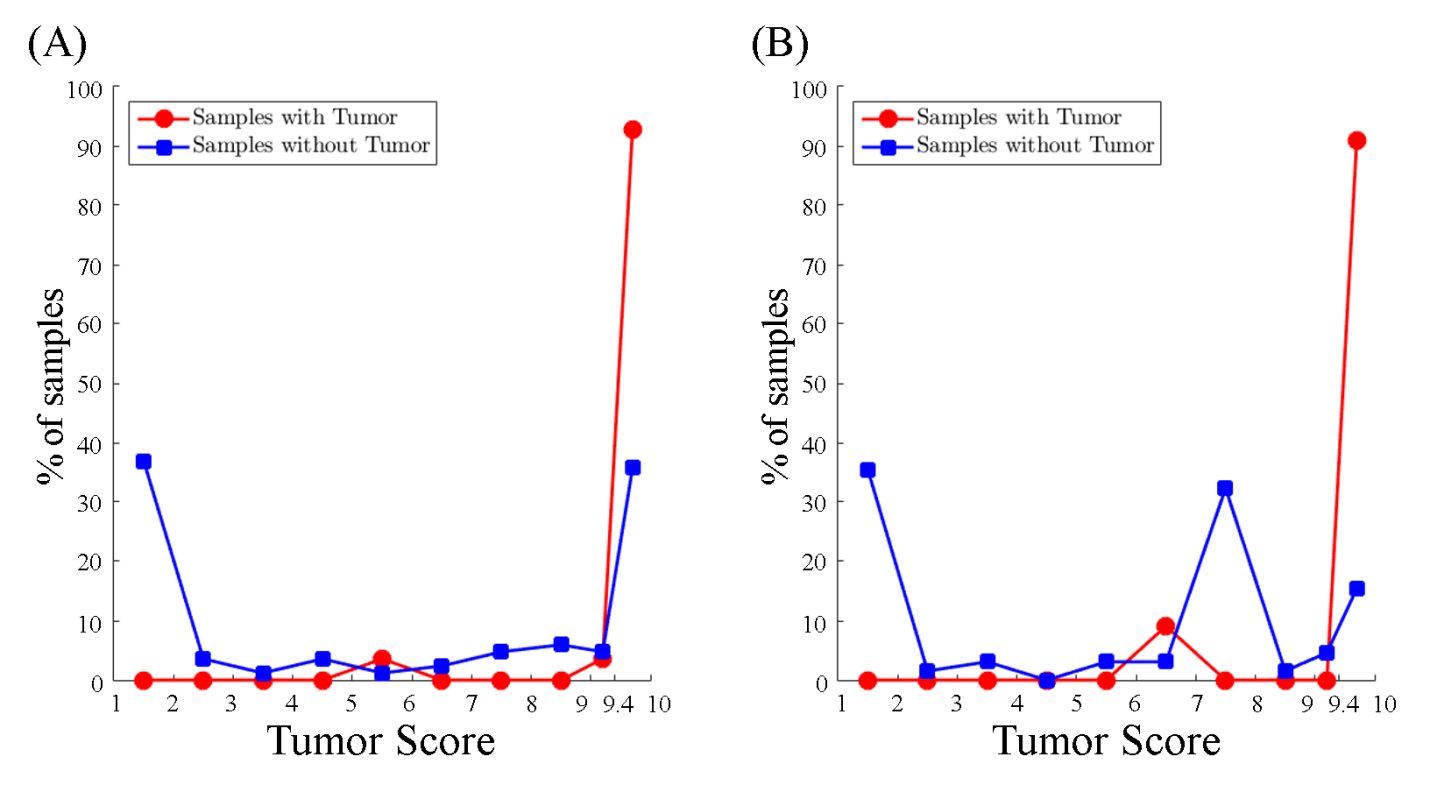


Figure S3: Histogram of maximum Tumor Scores for each sample in the (A) training set and (B) independent test set. Samples containing a segment with a Tumor Score greater than 9.0 were considered positive in this study.


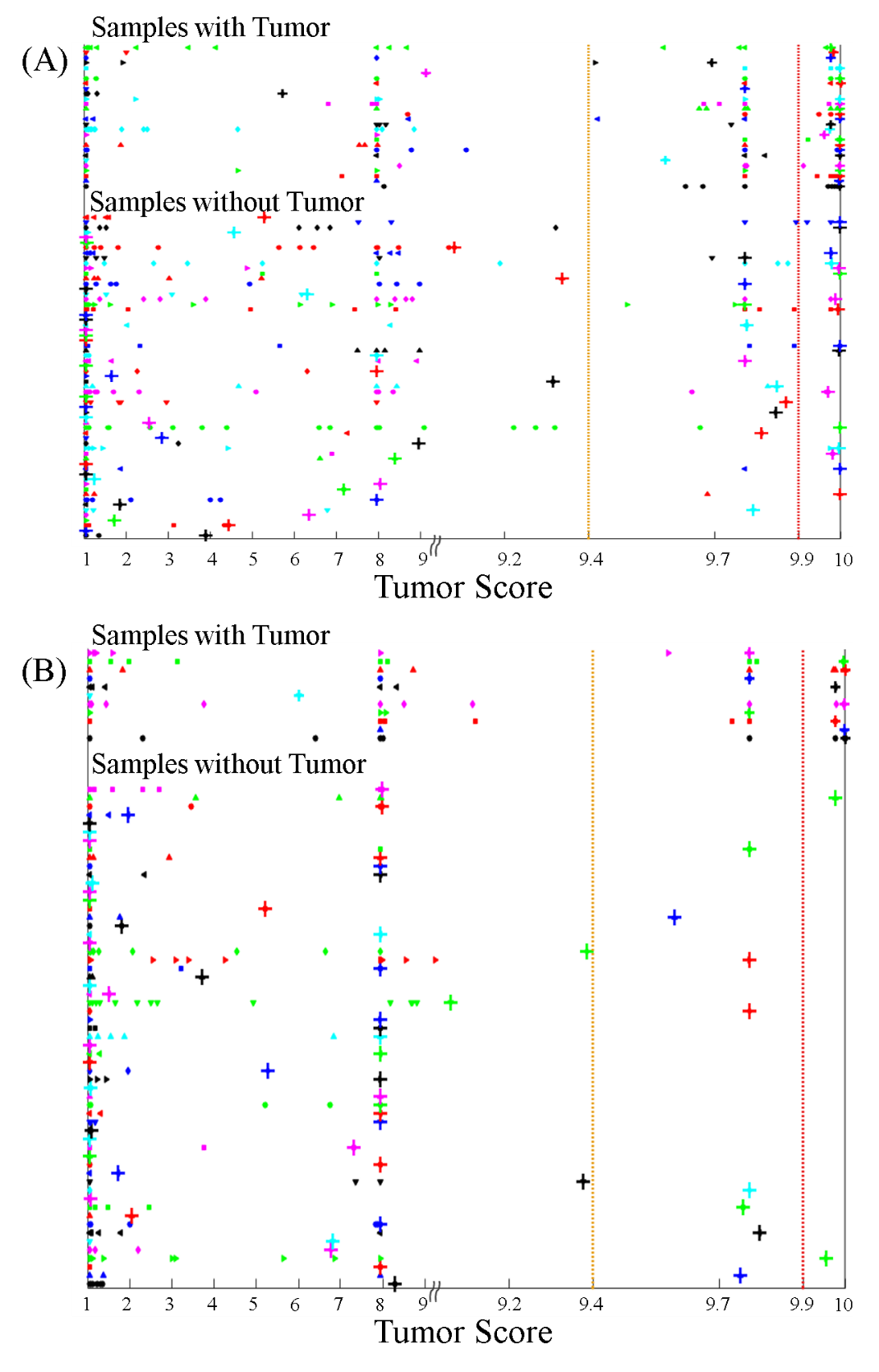


Figure S4: Tumor Scores for all segments in mastectomy tissue samples from the (A) training set and (B) independent test set. Each sample is represented by a shape/color combination, with each samples maximum Tumor Score marked with a “+.” (A) Thresholds set at Tumor Scores of 9.4 and 9.9 isolate tumor containing segments in samples from the training set with high sensitivity (96% of samples with tumor have a segment Tumor Score greater than 9.4) and high specificity (25% of samples without tumor have a segment with Tumor Score greater than 9.9). (B) Using the maximum Tumor Score for each sample in the independent test set, the Moderate Risk threshold at 9.4 yields 91% sensitivity and 83% specificity, while the High Risk threshold at 9.9 yields 64% sensitivity and 97% specificity.


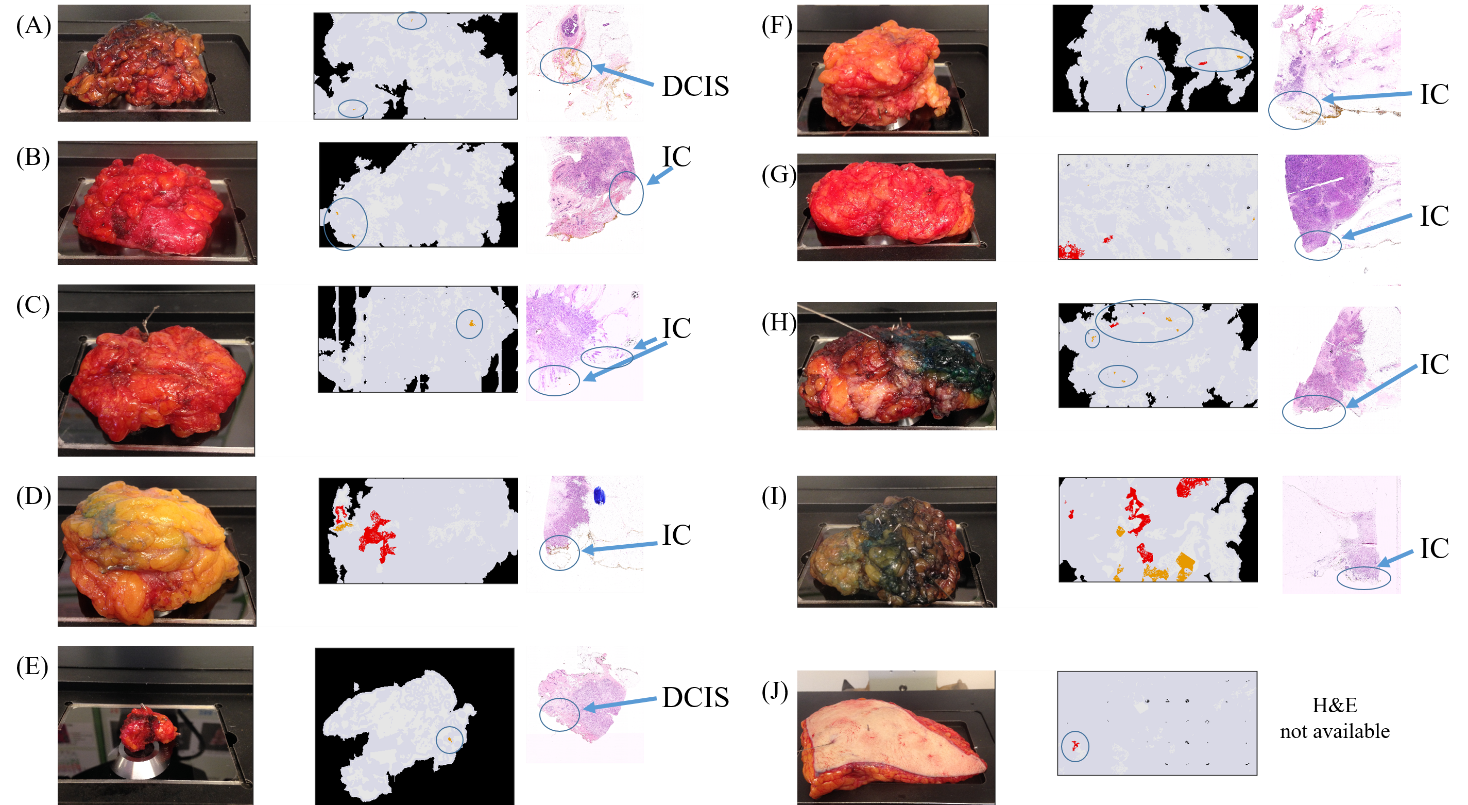


Figure S5: All BCS specimens with positive margins detected by MSH and confirmed by histopathology. Each MSH diagnosis image measures 64.8 x 32.4 mm^2^ and each H&E image measures 15 x 15 mm^2^. Circles mark the region of tumor intersecting the area measured by MSH (yellow ink). Some H&E sections were unavailable due to ongoing diagnostic procedures. DCIS = ductal carcinoma *in situ*, IC = invasive carcinoma.


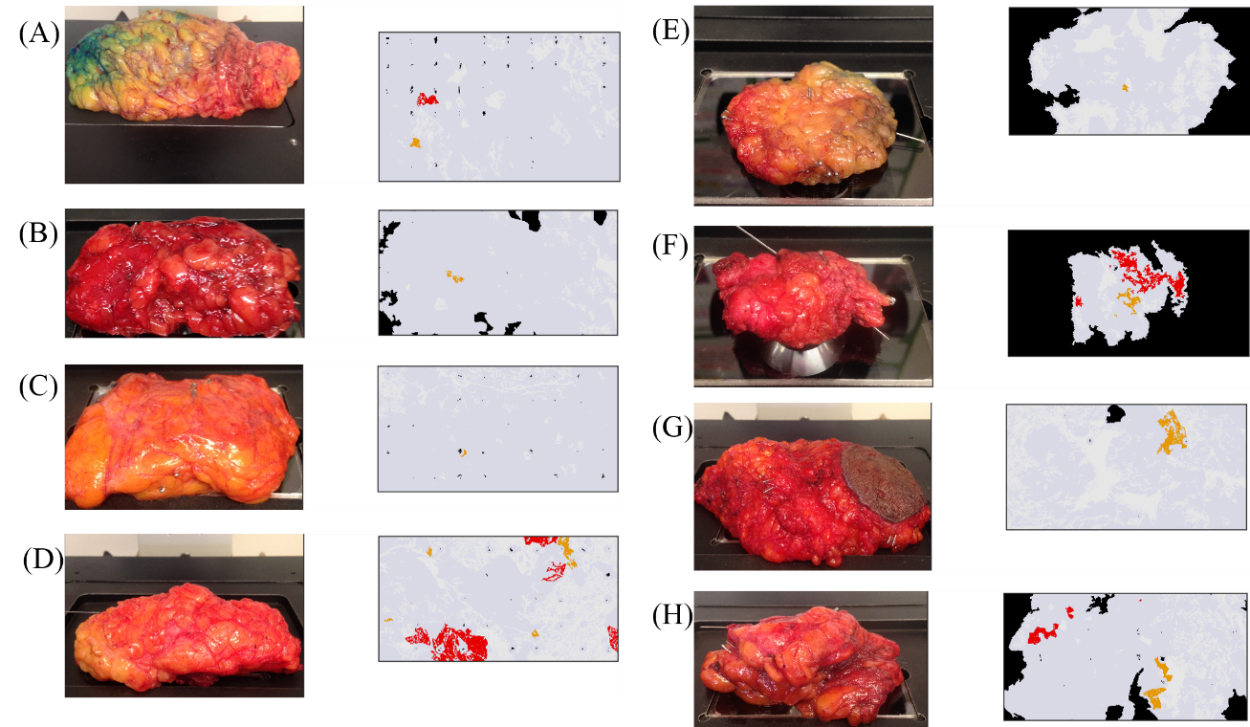


Figure S6: All BCS specimens for which MSH detected positive margins but no tumor was found at the surface in sections sampled by histopathology. Each MSH diagnosis image measures 64.8 x 32.4 mm^2^.


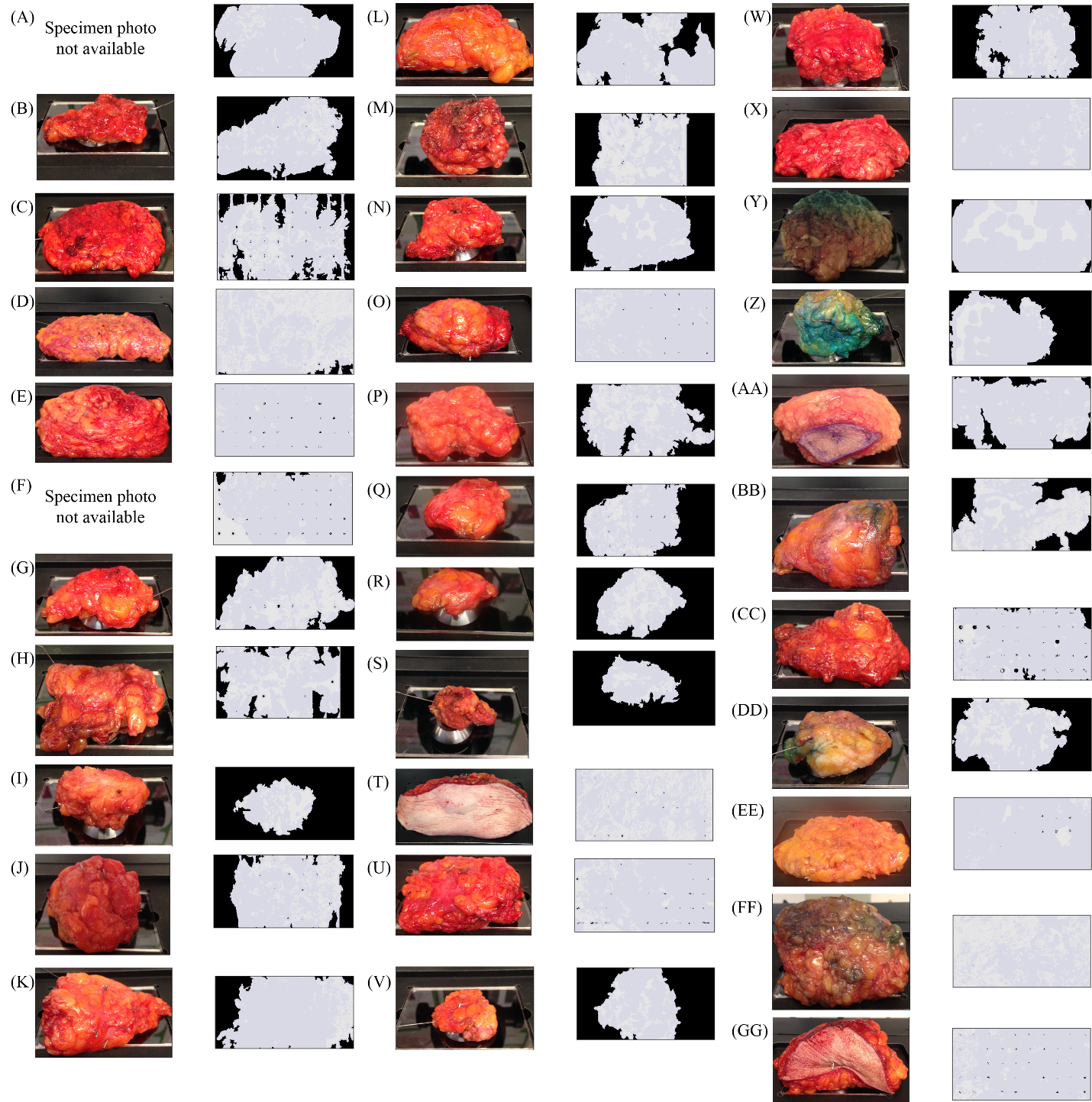


Figure S7: All BCS specimens for which the measured surface was diagnosed as clear by both MSH and histopathology. Each MSH diagnosis image measures 64.8 x 32.4 mm^2^.
